# The use of a mini-κ goniometer head in macromolecular crystallography diffraction experiments

**DOI:** 10.1107/S0907444913003880

**Published:** 2013-06-15

**Authors:** Sandor Brockhauser, Raimond B. G. Ravelli, Andrew A. McCarthy

**Affiliations:** a European Molecular Biology Laboratory (EMBL), 6 Rue Jules Horowitz, 38042 Grenoble, France; b Unit of Virus Host-Cell Interactions, UJF–EMBL–CNRS UMI 3265, 6 Rue Jules Horowitz, 38043 Grenoble, France; c Leiden University Medical Center (LUMC), PO Box 9600, 2300 RC Leiden, The Netherlands

**Keywords:** kappa goniometer, crystal alignment, data-collection strategies

## Abstract

Most macromolecular crystallography (MX) diffraction experiments at synchrotrons use a single-axis goniometer. This markedly contrasts with small-molecule crystallography, in which the majority of the diffraction data are collected using multi-axis goniometers. A novel miniaturized κ-gonio­meter head, the MK3, has been developed to allow macromolecular crystals to be aligned. It is available on the majority of the structural biology beamlines at the ESRF, as well as elsewhere. In addition, the *Strategy for the Alignment of Crystals* (*STAC*) software package has been developed to facilitate the use of the MK3 and other similar devices. Use of the MK3 and *STAC* is streamlined by their incorporation into online analysis tools such as *EDNA*. The current use of *STAC* and MK3 on the MX beamlines at the ESRF is discussed. It is shown that the alignment of macromolecular crystals can result in improved diffraction data quality compared with data obtained from randomly aligned crystals.

## Abstract 2

Hardware and software solutions for MX data-collection strategies using the EMBL/ESRF miniaturized multi-axis goniometer head are presented.

## 1. Introduction   

**Figure 1:**
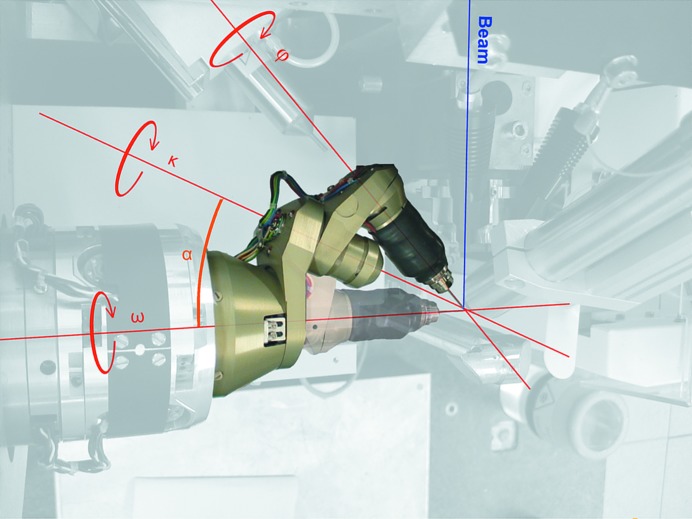
The EMBL/ESRF mini-κ goniometer head (MK3) as mounted on the MD2M diffractometer on ID14-4 in a ‘closed’ and in a fully ‘open’ conformation, highlighting the three rotational axes and the α angle.

A successful X-ray structural determination relies on the accurate measurement of diffraction intensities. One of the fundamental steps in an oscillation-based X-ray diffraction experiment (Arndt & Wonacott, 1977 ▶) is the precise alignment of the crystalline sample with the X-ray beam. In crystallo­graphy many types of goniometers have been developed, but kappa-based goniometers (κ-goniometers) are by far the most common. κ-Goniometers typically consist of three axes, each mounted on the other (Fig. 1 ▶). In full κ-goniometers the ω axis is perpendicular to the X-ray beam, the κ axis usually makes an α angle of ∼50° to ω, and a centred crystalline sample can be rotated around a final ϕ axis. Such κ-goniometers maximize the reciprocal-space coverage and allow all reflections to be accurately measured. Indeed, nearly all of the commercial X-ray diffractometers sold today have such a geometry. However, while the use of κ-goniometers is widespread in crystallography as a whole, they are seldom found on synchrotron-based macromolecular crystallography (MX) beamlines. There are various reasons for this, but the primary ones are their inherent large size and positional inaccuracy, which impinges on the limited sample-environment space available on MX beamlines and the minimum crystal size that can be measured.

While these restrictions have primarily limited their use in MX, the experimental advantages of using κ-goniometers for diffraction-based experiments have never gone away. These include aligning an evenfold crystallographic axis with the rotational axis so that Bijvoet mates can be collected on the same image for a more accurate measurement of the anomalous differences in experimental phasing protocols. Such goniometers also allow the collection of diffraction data from two or more orientations of the same crystal, providing a better ‘real redundancy’ than conventional single-orientation strategies by changing the path that equivalent reflections take (Debreczeni *et al.*, 2003 ▶). In addition, an experimental phasing protocol incorporating the anisotropy of anomalous scattering contributions from the intrinsic polarization of synchrotron radiation has been reported (Bricogne *et al.*, 2005 ▶; Schiltz & Bricogne, 2008 ▶). This protocol particularly benefits from the ability to align crystals (Schiltz & Bricogne, 2010 ▶) and can supplement the phase information available. One can also align specific crystallographic axes to experimentally determine an ambiguous space group, which is often useful in molecular-replacement protocols. Similar or even more sophisticated orientations can be used to minimize the total oscillation range needed, thus mitigating the detrimental effect of radiation damage. The manual manipulation of crystals required during crystal fishing and vitrification can often result in either intrinsic or induced mechanical crystal defects, which in favourable cases can be avoided by alignment strategies. Lastly, one can also purposefully mis-align a crystal to reduce the ‘blind zone’ and increase the data completeness (Dauter, 1999 ▶). These have all led to a renewed interest in κ-­goniometers, especially with ever more challenging structural determinations requiring the best-quality diffraction data possible for their ultimate success. Indeed, some modern MX beamlines, such as PROXIMA-1 at SOLEIL, are now equipped with full κ-goniometers, allowing a crystal to be aligned with some physical restrictions owing to the beamline environment.

However, one can also envisage a miniaturized κ-gonio­meter instrument that would both reduce the size and increase the positional accuracy when compared with a full κ-gonio­meter. In Grenoble we decided to develop such an instrument. This resulted in a mini κ-goniometer head version 3 (MK3) that is fully compatible with the MD2 and MD2M suite of diffractometers installed on many MX beamlines (Cipriani *et al.*, 2007 ▶; Brockhauser *et al.*, 2007 ▶). Indeed, similar miniaturized κ-goniometer instruments have also been developed elsewhere (Wang *et al.*, 2008 ▶; Shi *et al.*, 2006 ▶; Rosenbaum & Westbrook, 1997 ▶; Rosenbaum *et al.*, 2006 ▶). Four MK3s are now permanently installed and available on the EMBL–ESRF Joint Structural Biology Group beamlines at the ESRF. In order to further facilitate their routine use, the *STrategy for Aligned Crystals* (*STAC*) software suite has been developed. Here, we describe the crystal-alignment features of the MK3 using *STAC* as well as some of our recent results.

## 2. Methods   

### 2.1. The MK3 goniometer head   

All of the current MX beamlines at the ESRF are equipped with MD2 or MD2M diffractometers (Nurizzo *et al.*, 2006 ▶; McCarthy *et al.*, 2009 ▶; Flot *et al.*, 2010 ▶; de Sanctis *et al.*, 2012 ▶). These instruments allow the precise alignment of a crystalline sample with a small X-ray beam and have a measured sphere of confusion (SOC) of approximately 1 µm. Instead of implementing a full κ-goniometer system, a miniature crystal-orientation device with a limited κ-range was designed to minimize potential collisions with other devices. This resulted in the MK3 (Fig. 1 ▶), an easily exchangeable goniometer head that is fully compatible with MD2/MD2M diffractometers and SC3 sample changers (Cipriani *et al.*, 2006 ▶). The mounting of an MK3 on the MD2/MD2M currently results in an increase in the SOC to approximately 4 µm. The exchangeability allows the mounting of ancillary devices, such as a microspectro­photomoter (McGeehan *et al.*, 2009 ▶), which require additional space or the exchange to another goniometer head for plate screening (H. Belrhali, personal communication). There are now 22 MK3s distributed at ten synchrotron facilities worldwide (Table 1 ▶).

As with most κ-goniometers, the MK3 has the ϕ axis mounted on a κ axis, which is limited in range between −10 and 240°. The MK3 is subsequently mounted on the data-collection axis (or ω axis) of the MD2/MD2M, with an angle (α) of 24° between the κ and ω axes (Fig. 1 ▶). While this reduction in the α angle reduces the theoretical reciprocal space covered by the MK3 to 47%, it is a collision-free device. Traditional three-axis κ goniometers have the sample-centring stage mounted on the final ϕ axis so that all three axes intersect at a unique point, thus ensuring that the sample remains centred during an orientation procedure. Contrary to this, the MK3 uses the MD2/MD2M centring stage located before the κ and ϕ axes. Such inverse-κ systems consequently mean that the sample has to be re-centred after moving the κ or ϕ angle. To enable the use of such devices by maintaining the sample in the X-ray beam and cryostream, an elegant and automatic translation-correction protocol has been developed (Brock­hauser *et al.*, 2011 ▶). In addition, we use the ω axis of the MD2/MD2M for data collection as the ϕ axis of the MK3 is not sufficiently accurate.

### 2.2. *STrategy for Aligned Crystals* (*STAC*)   

**Figure 2:**
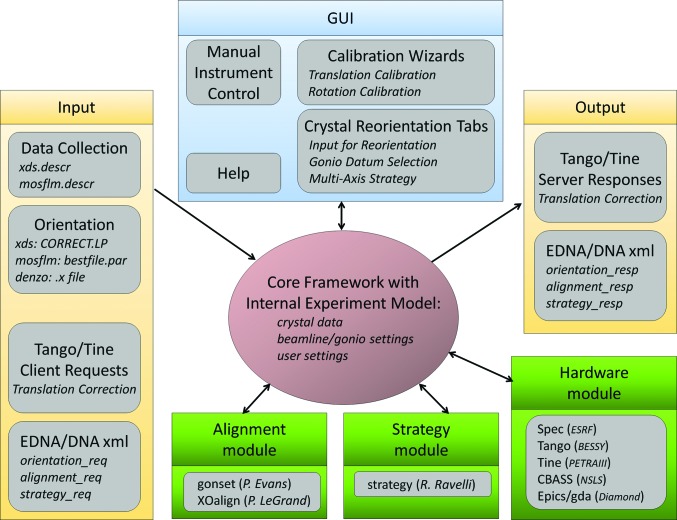
A schematic representation of *STAC*. The core framework, which can manage input or output information flow (yellow) from/to various external software solutions, is shown in magenta. The GUI interface, with the various ‘tabs’, is shown in blue. The service modules for alignment and strategy calculation as well as for hardware access are shown in green.

In order to facilitate the use of the MK3 on synchrotron-based MX beamlines, a standalone Java-based software suite, *STrategy for Aligned Crystals* (*STAC*), was developed. The program is based on object-oriented (OO) design patterns and takes advantage of the cross-platform support of the Java Virtual Machine for execution on different operating systems. The graphical user interface (GUI) elements can also be easily implemented in other Java applications or web applets, thus facilitating their integration with synchrotron data-collection programs such as *automateD collectioN of datA* (*DNA*) or *Generic Data Acquisition* (*GDA*). The software design has been guided by a few additional constraints: the ability to embed existing scientific codes and applications, flexibility for easy extension with new features or variants, parallel computational support, asynchronous user-request management and support for a variety of hardware implementations found at synchrotrons. Because of all these constraints, the software design does not follow a simple linear program structure. Rather, it provides a ‘framework’ for implementing and incorporating services. This framework groups the various services into ‘modules’ with defined interfaces, as illustrated in Fig. 2 ▶. The implementation freedom of the OO interface guarantees flexibility by accepting alternative solutions and inheritance for efficient coding. The plug-in architecture of the modules provides easy run-time access to the implemented services and their variants.

**Figure 3:**
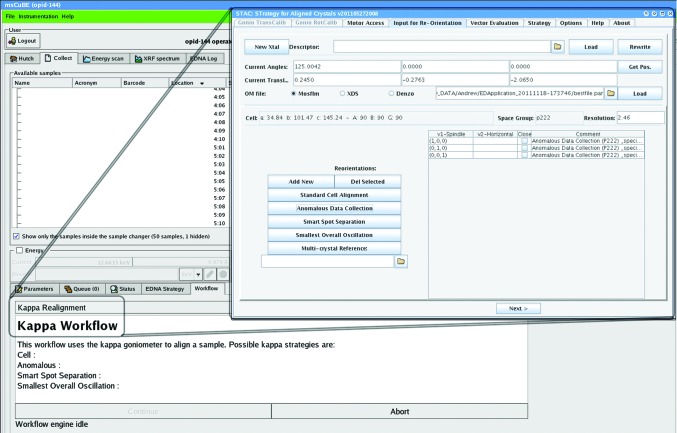
Screenshot of *STAC*, illustrating how it can be used in either a standalone or a server application mode, such as through the workflow tab in *MxCuBE*. The *STAC* GUI shows the ‘Input for Re-Orientation’ tab with the ‘Anomalous Data Collection’ options for a *P*2_1_2_1_2_1_ crystal system.

Currently, three modules have been defined: an alignment module for alignment calculation, a strategy module for multi-sweep multi-axis data-collection strategy calculations and a hardware module for accessing the underlying kappa instruments such as the MK3. These are illustrated in Fig. 2 ▶. Since the implementation of these modules embeds existing scientific codes, the interface also has a declaration method to credit the original author contributions. Current implementations of the alignment module include *GONSET* from P. Evans (MRC-LMB, Cambridge) and the Python-based *XOalign* from P. LeGrand (PROXIMA 1, SOLEIL). The Strategy module is implemented using *STRATEGY* (Ravelli *et al.*, 1997 ▶). A configuration file is used to set up the default behaviour of the application by activating and setting up the services as instances of the defined module implementations. The framework automatically manages the whole lifecycle of any service requests, including creation, maintenance (progress monitoring and error handling), control (starting and abortion) and response handling. It supplies a thread-safe environment for asynchronous execution of processes and automatically synchronizes the output to the GUI. *STAC* can be run as a standalone GUI communicating with relevant beamline-control motors, as shown in Fig. 3 ▶, or in a device–server mode. When launched without the GUI front end, the program reads the input ‘from’ and writes the output ‘to’ various xml files using the *DNA* data model. For proper calculations *STAC* must have a calibrated kappa instrument model. Hence, the GUI provides easy-to-use wizards for the rotational calibration needed for any alignment calculation and the translational calibration needed to maintain the sample in the X-ray beam and cryostream when changing the orientation on an inverse-κ system (Brock­hauser *et al.*, 2011 ▶).

### 2.3. *STAC* alignment procedures   

A successful alignment procedure depends on two conditions: the position of the MK3 and sample-centring motors and the initial crystal orientation. The former can be retrieved from the beamline-control software, while the latter requires a simple crystallographic characterization. *Enhanced DNA* (*EDNA*) is an online diffraction-characterization and data-collection strategy algorithm that is now widely used (Incardona *et al.*, 2009 ▶). We therefore recommend that *EDNA*, or a similar characterization program such as *RAPD*, is used to determine the initial orientation of a crystal. *STAC* can also read all of the commonly used crystallographic orientation-matrix file formats, including those from *XDS* (Kabsch, 2010 ▶) and *MOSFLM* (Leslie & Powell, 2007 ▶). The relevant information can then be retrieved by *STAC* for the calculation of the alignment protocols in the ‘Input for Re-Orientation’ tab (Fig. 3 ▶). At the ESRF, we have now implemented the use of workflows into the beamline-control software *MxCuBE* (Gabadinho *et al.*, 2010 ▶) to facilitate the routine use of crystal-alignment procedures (Brock­hauser *et al.*, 2012 ▶). A number of standard options, as shown in Fig. 3 ▶, are available and are described below. *STAC* also contains a simple option to tilt crystals using an on-axis viewer. Note that this is only valid if the κ axis is initially in the plane of the beam and spindle. This option is particularly useful for increasing the data complete­ness by minimizing the ‘blind’ zone of fortuitously aligned crystals (Dauter, 1999 ▶).

#### 2.3.1. Standard cell alignment   

The standard cell-alignment protocol is the most commonly used and results in the creation of a table containing all of the relevant orientation possibilities for a reciprocal cell axis alignment. The results are displayed in the ‘Vector Evaluation’ tab and a message window will appear to inform the user which orientations are not allowed owing to the limitations of the MK3. Depending on the space-group symmetry, several alternative triplets (ω, κ and ϕ) are calculated and displayed for aligning one reciprocal cell axis along the ω axis and a second in the plane of the X-ray beam and ω, resulting in two possible ω solutions separated by 180°. In cases where the unit-cell angles are 90°, the same κ and ϕ angles with a 90° separation in ω result in the alignment of one reciprocal cell axis along the spindle (*e.g.* c*) and different axes in the plane (*e.g.* a* or b*). If the desired orientation is not possible, one can simply return to the previous tab, tick the ‘close’ box and rerun the *STAC* algorithm. The tick-box option is always available and will return an alignment vector that brings the selected reciprocal axis as close as possible to the spindle or ω axis, resulting in one or both of the other reciprocal axes moving into the diffraction condition. This re-orientation option is particularly useful for experimentally measuring the systematic absences along each crystallographic axis and can help in the identification of the correct space group.

#### 2.3.2. Anomalous data collection   

The anomalous data-collection protocol results in the creation of a table containing all of the possible orientations that enable the collection of Bijvoet mates (a reflection and the Friedel pair of its symmetry equivalent, *e.g. hkl* and 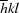) on the same image by aligning an evenfold-symmetry axis perpendicular to the spindle axis, as shown in Fig. 3 ▶ for a primitive ortho­rhombic space group. This procedure launches a *STAC* algorithm that returns all such orientations, as well as a message window informing the user about those that are not possible owing to MK3 design limitations. This alignment option results in more accurately measured anomalous differences, as the Bijvoet mates are measured simultaneously in dose. This option can therefore help to mitigate against the detrimental effects of convoluting anomalous differences with radiation-damage-induced intensity changes (Ravelli *et al.*, 2005 ▶).

#### 2.3.3. Smart spot separation   

Thanks to the advances in recombinant protein production, many larger and more complex biological systems are becoming amenable to study by X-ray crystallography. Owing to their size, such systems normally crystallize with large unit cells and their corresponding diffraction patterns can sometimes be hard to resolve, even on the most modern synchrotron-based MX beamlines that are equipped with large CCD or pixel-array detectors. This normally manifests itself as overlapping reflections that are hard to deconvolute and typically occurs when samples with an exceptionally long cell axis are not optimally oriented during the crystal-fishing process. While this problem can be exaggerated by a large mosaicity, it is not considered in the current algorithm, which only concentrates on the orientation effects discussed by Dauter (1999 ▶). To enable users to collect the best possible data from such crystal systems, *STAC* provides an algorithm that returns an optimal orientation for maximizing the separation of reflections. Selection of this option results in the creation of a table with orientations where the densest direction of the reciprocal space, which is most commonly the longest unit-cell axis, is aligned close to the spindle. The precise alignment of the cell axis along the spindle would normally result in a blind zone (Dauter, 1999 ▶). Therefore, to avoid this scenario the crystal orientation is tilted slightly to ensure that the maximum completeness is attained for the desired resolution.

#### 2.3.4. Smallest overall oscillation   

All experimental X-ray diffraction measurements on biological systems are sensitive to radiation damage. Many protocols and algorithms have been devised to try and mitigate against these effects, but one of the simplest methods is to reduce the total oscillation range required for the desired completeness. The algorithm used in *STAC* was first implemented in *STRATEGY* (Ravelli *et al.*, 1997 ▶) and has been so widely used that we included this option in a ‘user-friendly’ manner. The selection of this option results in the creation of a table that suggests several alternative orientations for completeness targets of >98%. For each of these orientations the required minimum collection wedge is also displayed.

#### 2.3.5. Multi-crystal reference   

The last option available in *STAC* enables the reproduction of a previously used crystal orientation. Hence, the re-collection of the same crystallo­graphic data set from a new crystal can be performed, or data collection from a previous crystal can be continued using the new crystal, provided that they are isomorphous. This option requires the orientation matrix of the previous crystal to be loaded into *STAC*. This information is then combined with the current crystal description in *STAC* during the orientation calculation, returning the new goniometer settings to be applied. We assume that a data-collection strategy has already been calculated and started with the previous crystal but that it could not be completed, for example owing to radiation damage. The returned ω value must therefore be ‘offset’ to account for the previous data wedge, and the next data collection continued from this new starting angle. This option enables the collection of a complete data set from a number of isomorphous crystals. While not routinely used at present, the availability of a high-precision goniometer equipped with a MK3 with a much reduced SOC (<1 µm) and the observation that microcrystals fished from a single loop are often more isomorphous than those from different loops (Giordano *et al.*, 2012 ▶) should encourage the use of more complicated multi-crystal data-collection strategies on microcrystals.

## 3. Experimental results from crystal-alignment strategies   

The ultimate success of an MX experiment is dependent on the accurate measurement of a complete set of reflections. The collection of such X-ray diffraction data from aligned crystals should improve the data quality achievable and should be considered in all data-collection strategies. To provide such an option, the MK3 and its associated *STAC* software suite have been made available on most of the MX beamlines at the ESRF since 2008. We now routinely use the MK3 for crystal alignment and actively encourage its use. More users are making use of this device and a recent search in ISPyB (Delagenière *et al.*, 2011 ▶) revealed that alignment strategies were used in 6.7% of all data collections on ID14-4 in 2011 (Brockhauser *et al.*, 2012 ▶). Below, we outline just some of our recent experimental results, which illustrate that the data quality can be improved using crystal-alignment strategies. Such devices can also enable the inclusion of additional information from the inherent polarization effect on synchrotron sources in more complex phasing calculations (Schiltz & Bricogne, 2010 ▶).

### 3.1. A high-energy S-SAD phasing experiment   

**Figure 4:**
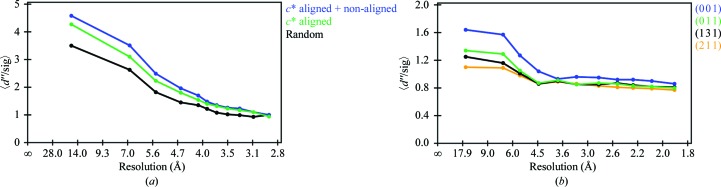
*SHELXC* results highlighting the increase in the anomalous signal that can be achieved by measuring an aligned crystal along a twofold axis: (*a*) for a high-energy (*E* = 13.2 keV) bovine trypsin S-SAD phasing experiment, (*b*) for an Se-SAD phasing experiment.

The ID14-4 MX beamline (McCarthy *et al.*, 2009 ▶) at the ESRF is equipped with an ADSC Q315r Mosaic CCD detector (ADSC, Poway, California, USA) and has a maximum peak intensity of 4.5 × 10^12^ photons s^−1^ using a 100 × 100 µm slit size at 13.2 keV or 0.9393 Å. At this energy the theoretical Bijvoet ratio 〈|Δ*F* ^±^|〉/〈|*F*|〉 for crystals of native bovine trypsin, which contains 14 S atoms (12 cysteines in six disulfide bridges and two methionines) and one Ca^2+^ ion, is estimated to be ∼0.8% (Dauter, 2006 ▶; Hendrickson & Teeter, 1981 ▶). This makes an S-SAD phasing experiment on such a system quite challenging at this energy. We therefore decided to experimentally determine whether a crystal-alignment strategy could facilitate the *ab initio* phasing of bovine trypsin at 13.2 keV. For this experiment, we collected four independent 360° data sets at two crystal positions on a large (200 × 200 × 1000 µm) rod-shaped crystal (unit-cell parameters *a* = 54.3, *b* = 58.1, *c* = 66.8 Å) with *P*2_1_2_1_2_1_ space-group symmetry. Each data set was collected at a different crystal orientation: a data set with c* aligned with the rotation axis and three other arbitrary data sets (Table 2 ▶) using a 0.1 s exposure and 8% transmission. This resulted in a photon flux of 1.2 × 10^11^ photons s^−1^ at the sample position and a total absorbed dose, as calculated using *RADDOSE* (Murray *et al.*, 2004 ▶; Paithankar & Garman, 2010 ▶), of 150 kGy per data set. All data were processed and scaled using the *XDS* suite (Kabsch, 2010 ▶) and *SCALA* (Evans, 2006 ▶), are of very high quality (Table 2 ▶) and were isomorphous (*R*_iso_ of between 2.3 and 3.5%). We then compared the resulting anomalous signal-to-noise ratios calculated using *SHELXC* and visualized with *HKL*2*MAP* v.0.3 (Pape & Schneider, 2004 ▶). As expected, the c*-aligned orientation has a much larger signal compared with the other orientations (Fig. 4 ▶ *a*). This observation is consistent with the fact that we could unambiguously determine the correct substructure for the aligned data set with *SHELXD* (Sheldrick, 2008 ▶), while attempts with all of the other alignments were unsuccessful (Table 2 ▶). A few rounds of solvent flattening with *SHELXE* using the autotracing routine (Sheldrick, 2010 ▶) resulted in a clearly interpretable electron-density map and 194 polyalanine residues were correctly built out of a total of 223. This is despite the c*-aligned orientation having a marginally lower complete­ness (Table 2 ▶) owing to the blind zone (Dauter, 1999 ▶). Even better results were obtained upon merging the aligned data set with one or more of the other data sets. This is consistent with previous observations that the merging of diffraction data collected from different crystal orientations results in a higher ‘real redundancy’ (Debreczeni *et al.*, 2003 ▶). This strategy can be used to minimize any systematic errors acquired during the measurement of a highly redundant diffraction data set from a single crystal orientation.

### 3.2. A selenomethionine SAD phasing experiment   

The improvement that can be achieved by aligning a crystal for an *ab initio* phasing experiment was again demonstrated by solving the structure of a domain of a protein involved in piRNA biogenesis using a selenomethionine-based SAD phasing experiment (Cora *et al.*, unpublished results). This domain of ∼15 kDa crystallized in space group *P*2_1_2_1_2_1_, with unit-cell parameters *a* = 35.2, *b* = 100.8, *c* = 146.3 Å, and contained four molecules in the asymmetric unit with a solvent content of 41%. Despite the large theoretical Bijvoet ratio 〈|Δ*F* ^±^|〉/〈|*F*|〉 of ∼9% (Hendrickson & Teeter, 1981 ▶), it turned out to be a non-straightforward phasing experiment. For the best crystal, we collected two consecutive data sets at the selenium fluorescence peak (12.661 keV) from a single position in two different crystal orientations. The first was collected with the c* axis aligned with the rotation axis and the second was collected in a random orientation. Both data sets were optimized to minimize the ϕ range needed to measure a complete set of anomalous differences for a particular orientation. We used a 60 × 60 µm slit size to match the crystal size, 30% transmission and a 0.3 s exposure time. This resulted in a photon flux of 3.6 × 10^11^ photons s^−1^ at the sample position and a total absorbed dose, as calculated using *RADDOSE* (Murray *et al.*, 2004 ▶; Paithankar & Garman, 2010 ▶), of 2.9 MGy per data set. All data were processed and scaled using the *XDS* suite (Kabsch, 2010 ▶) and *SCALA* (Evans, 2006 ▶), are of very high quality (Table 3 ▶) and have an *R*_iso_ of 9.7%.

**Figure 5:**
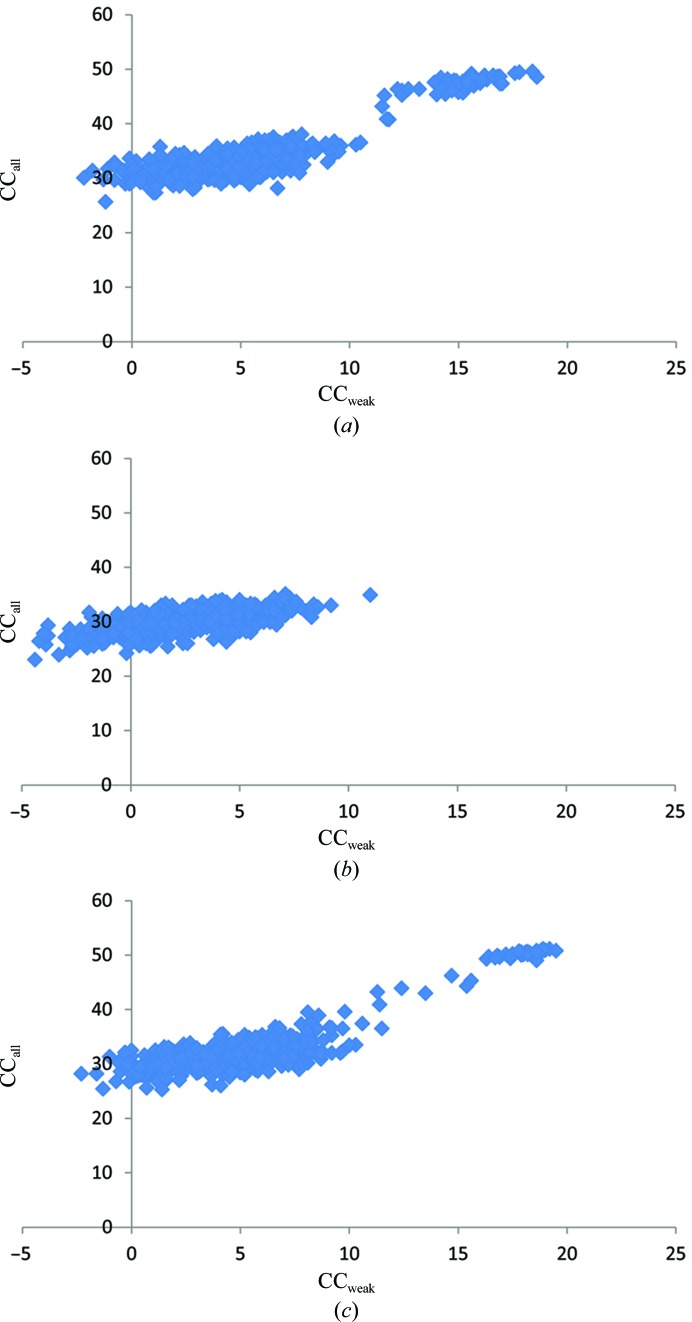
CC_all_ *versus* CC_weak_ *SHELXD* plots for the three data sets in Table 3 ▶: (*a*) c* -aligned data set, (*b*) non-aligned data set, (*c*) merged data set.

The first thing that we noticed was that the aligned data set contained 2128 (or ∼20% of the total) more Bijvoet pairs compared with the unaligned data set. Perhaps more important is the fact that these reflections also had a larger anomalous signal (Table 3 ▶ and Fig. 4 ▶ *b*). Similar to the high-energy S-­SAD case described above, we could easily find the heavy-atom sites using the aligned data set alone but not the non-aligned data set (Fig. 5 ▶). As we could not obtain good phases with *SHELXE* (Sheldrick, 2010 ▶), we turned to *SHARP* (de La Fortelle & Bricogne, 1997 ▶). The experimental phases calculated using the SAD procedure in *SHARP* (de La Fortelle & Bricogne, 1997 ▶) were further improved using the density-modification package *SOLOMON* (Abrahams & Leslie, 1996 ▶) and resulted in clearly interpretable electron-density maps. Since the randomly oriented data set was collected after the aligned data set, we cannot rule out that radiation damage also contributed to the differences. However, we believe it to be a moderate effect given the low dose used per data set, the small decrease in 〈*I*/σ(*I*)〉 and the minor increase in the Wilson *B* factor (Table 3 ▶). In addition, the combined data set resulted in a better experimental map that was used as the starting point for subsequent model building and refinement.

### 3.3. Space-group determination   

The success of any structure determination depends on assigning the correct space group. Most X-ray diffraction data measurements at synchrotron sources preclude the measurement of axial reflections along one or more of the crystallo­graphic axes. In the absence of such experimental information it is often necessary to test multiple space groups in a translational search. However, the ability to easily align a crystal with the MK3 and *STAC* while it is still mounted can now be used to bring another crystallographic axis into the diffraction condition to enable the measurement of all possible systematic absences. It can even be carried out after the initial data set has been collected and is something that we now routinely use and recommend to all users wishing to solve new structures.

**Figure 6:**
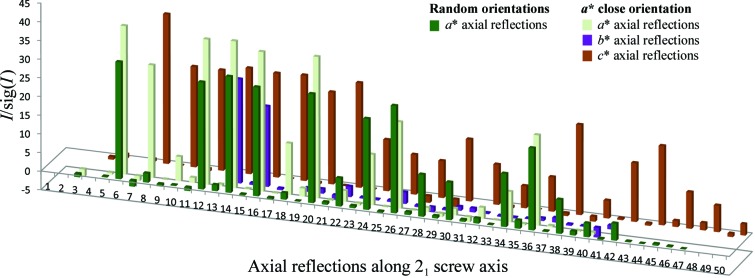
A plot of the axial reflections along a*, b* and c* highlighting how most of the systematic absences can be experimentally measured by collecting data at different orientations.

For example, we have recently crystallized a synthetic FAB–receptor complex. We screened several crystals from a single crystallization drop, but only one of these was of sufficient quality for the collection of data. Unfortunately, during the mounting process the crystal was orientated with the c* axis very nearly aligned along the data-collection axis, precluding the measurement of axial reflections along the c* axis (Fig. 6 ▶). We nevertheless collected an initial data set according to the *EDNA* strategy in this random orientation (100 images with a 1° oscillation range). In addition, no systematic absences could be measured along b* because it remained in a plane containing the X-ray beam and perpendicular to the spindle axis, and therefore did not cross the Ewald sphere in the data-collection range used. These reflections could have been measured had the rotation range been extended, but we had already reached the desired completeness. In order to experimentally measure the systematic reflections from b* and c* we decided to use *STAC* and the MK3 to orient the crystal with the a* axis as close as possible to the rotation axis (an a* alignment was not possible in this case). We then collected another full data set in this orientation (100 images with a 1° oscillation range), allowing us to measure enough systematically absent reflections so that we could unambiguously assign the space group as *P*2_1_2_1_2_1_ (Fig. 6 ▶). This is a very simple routine and while we did not use the second data set for the structural solution and refinement owing to radiation damage, it greatly simplified the molecular-replacement search.

### 3.4. Long axis   

It has been reported that around 57 full data sets are required for each PDB deposition (Chruszcz *et al.*, 2008 ▶). This rather large ratio is presumably a consequence of the various factors that can hinder successful structure solution. Such a high attrition rate is dependent on many factors such as the crystal quality and the need to collect many data sets for experimental phasing. Nevertheless, a large number of these experiments also fail because of poor experimental planning. The introduction of *EDNA* (Incardona *et al.*, 2009 ▶) on the MX beamlines at the ESRF has resulted in an improvement in the data-quality statistics at the ESRF, but there is clearly still scope for further improvement. X-ray-induced radiation damage (Ravelli & Garman, 2006 ▶) is widely recognized as a major factor hindering the success of most diffraction experiments, but there are many other common pitfalls, of which one is spot overlap (Dauter, 1999 ▶).

**Figure 7:**
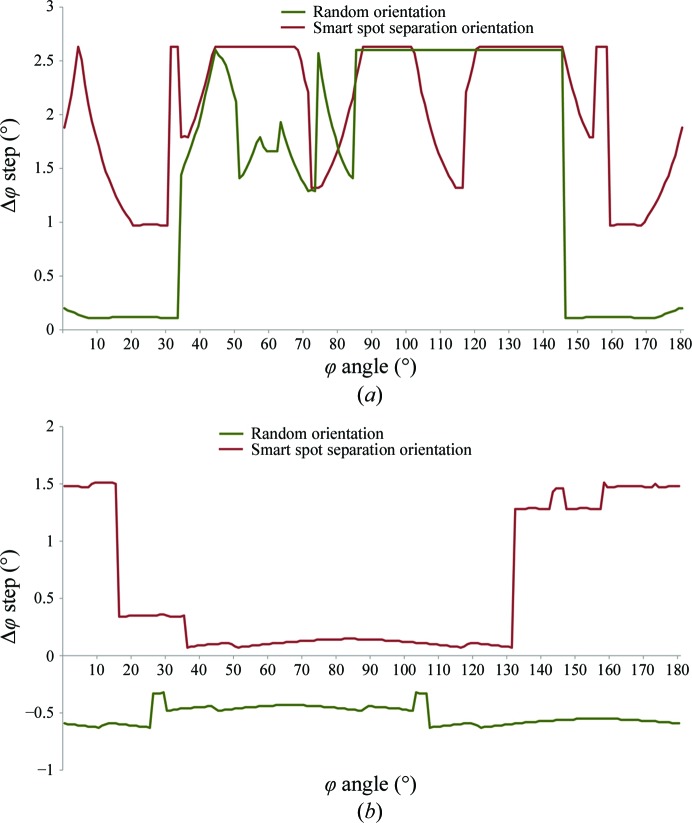
Plot of the maximal oscillation width possible to avoid overlapping spots as calculated using *BEST* (Bourenkov & Popov, 2010 ▶) (*a*) for a deliberately misaligned thaumatin test crystal at 1.4 Å resolution and (*b*) for a 70S ribosome test crystal at 3.8 Å resolution.

To best demonstrate how this hurdle can be overcome using the MK3, we performed two experiments. In the first experiment we deliberately misaligned a thaumatin test crystal, which crystallized in space group *P*4_1_2_1_2 with unit-cell parameters *a* = *b* = 58.1, *c* = 150.8 Å, in the sample loop during the mounting and vitrification process. This resulted in the longer c* axis lying nearly perpendicular to the data-collection axis. We then ran a simple *EDNA* characterization on ID14-4 at a maximal detector resolution of 1.4 Å. This was followed by a ‘smart spot separation’ using the kappa workflow routine (Brockhauser *et al.*, 2012 ▶) and another *EDNA* characterization at the κ angles suggested. The calculation of an optimal *EDNA* data-collection strategy was performed using *BEST* (Bourenkov & Popov, 2010 ▶), which combines an extensive diffraction analysis with the experimental conditions used for the characterization. One important data-collection parameter calculated is the optimal Δϕ necessary for minimizing the number of spot overlaps. This is also presented in an easy-to-interpret plot that is displayed to the user. Such a plot is shown in Fig. 7 ▶(*a*) for the initial ‘random’ orientation superimposed with a plot from the ‘smart spot separation’ orientation suggested by *STAC*. This comparison clearly shows how the maximal Δϕ recommended can be dramatically improved using ‘smart spot separation’. We also carried out a similar experiment using a 70S ribosome test crystal kindly supplied by the Ramakrishnan laboratory (Selmer *et al.*, 2006 ▶), which belonged to space group *P*2_1_2_1_2_1_ with unit-cell parameters *a* = 213.5, *b* = 456.9, *c* = 626.9 Å, using the recently installed PILATUS 6M detector on ID23-1 (Nurizzo *et al.*, 2006 ▶). Here, the maximal detector resolution was set to 3.9 Å and the results are shown in Fig. 7 ▶(*b*). This time *BEST* produces negative values for the Δϕ range in the random orientation, which is indicative of not finding a suitable Δϕ to avoid overlaps. This contrasts with the ‘smart spot separation’ routine, which returns a much improved spot separation, as observed by a remarkable increase in the maximal Δϕ possible.

### 3.5. Other possibilities   

Apart from the experimental results shown above, *STAC* and the MK3 have been used for several other applications. These include plate-shaped crystals, which are particularly prone to deformation during the manual fishing and vitrification steps. Such systems often diffract inhomogeneously; however, by using a small beamsize in combination with a grid or mesh scan the best part (or parts) of the crystal can be identified and used for eventual data collection (Bowler *et al.*, 2010 ▶). On MX beamlines with a larger beamsize one can also align the crystal and reduce the horizontal beamsize to minimize the deformed diffraction volume illuminated. This has been effectively used on ID14-4 (McCarthy *et al.*, 2009 ▶) and a similar approach can also be used to avoid other defects resulting from intentional or unintentional manipulation during crystal fishing. Another example is when users manage to mount a crystal nearly perfectly parallel in the loop either through design or by accident. Such a near-perfect alignment can result in a less complete data set being collected owing to the ‘blind zone’ (Dauter, 1999 ▶). In these situations, we advise users to slightly misalign the crystal using *STAC* and the MK3 so that a more complete data set can be obtained. Our last example is for biological systems that crystallize as large rod-shaped crystals with one crystal dimension significantly larger than the other two. Here, *STAC* and the MK3 provide the user with an opportunity to align the spindle with the largest crystal dimension. This allows the maximum diffraction volume of the crystal to be maintained in the X-ray beam. One can then divide the crystal into segments by adjusting the horizontal beamsize and systematically progressing along the crystal. Such a strategy allows one to mitigate against the detrimental effects of radiation damage (Zeldin *et al.*, 2013 ▶) and has been extensively used for the collection of many complete high-resolution ribosome data sets on ID14-4 (Schmeing *et al.*, 2009 ▶).

## 4. Discussion   

In this paper, we have described the alignment options currently available with an MK3 using *STAC* at the ESRF. A manual is available on the ESRF structural biology beamline webpages for help with such experiments. MX beamlines at other synchrotrons use a variety of different protocols. One example of this is the implementation of *STAC* in the RAPD data-analysis webserver on NE-CAT at the APS (J. Schuermann & F. Murphy, personal communication). At the ESRF we have recently developed a *Data Analysis WorkBench* (*DAWB*) to enable alignment strategies to be executed through *MxCuBE*. After the deployment of this integrated solution, a significant increase in the use of the MK3 on ID14-­4 (McCarthy *et al.*, 2009 ▶) was observed, increasing from about 6.7 to 13% in the last two months of user operation in 2011 (Brockhauser *et al.*, 2012 ▶).

Many experimental phasing methods exist for *ab initio* phase determination, but the most common and successful is the single-wavelength anomalous diffraction (SAD) method. The success or failure of this method is dependent on the accurate measurement of the Bijvoet mates to ensure that the best anomalous signal can be extracted. Several procedures to optimize these differences are often essential for its success, including measuring the Se *K*-edge X-ray fluorescence spectrum and collecting diffraction data at the peak determined by *CHOOCH* to maximize the *f*′′ contribution (Evans & Pettifer, 2001 ▶). Another factor to consider for a SAD phasing experiment is to ensure that the multiplicity of the data is sufficient for an accurate measurement of the anomalous signal. However, this must be carefully weighted against the fact that SAD phasing experiments can easily fail owing to radiation damage. It is therefore advisable to determine how many data are required and what dose a sample can take using advanced strategy-prediction programs such as *BEST* (Bourenkov & Popov, 2010 ▶). Theoretical simulations using *BEST* show that the anomalous signal can most accurately be measured by aligning a crystal (Brockhauser *et al.*, 2012 ▶). These results have now been validated here and we have shown two examples in which this can lead to the success or failure of the phasing experiment. We now routinely use *STAC* and the MK3 for all our phasing experiments and strongly advise our users to first collect a complete aligned data set followed by an unaligned data set. In our experience this results in the best experimental phases and ensures that the optimal data are collected first in case of detrimental radiation damage occurring in the second data collection. For example, this strategy was used to collect the anomalous data sets for the full-length and ΔCTD RIG-I structures used to verify the molecular-replacement solutions (Kowalinski *et al.*, 2011 ▶). Other known examples of anomalous data collected from aligned crystals include the structural solution of human mitochondrial mTERF in complex with DNA (Jiménez-Menéndez *et al.*, 2010 ▶) and the structure of an outer membrane complex of the type IV secretion system (Chandran *et al.*, 2009 ▶).

Several possibilities now exist for optimizing MX data-collection experiments using crystal-alignment strategies. These include the ability to minimize the total oscillation range necessary to help mitigate against radiation damage, the experimental measurement of systematic absences for space-group determination, the possibility of minimizing the number of overlapping reflections when a dense reciprocal-lattice plane crosses the Ewald sphere, the misalignment of nearly perfectly aligned crystals to minimize the blind zone and the ability to avoid defects or maximize the illuminated diffraction volume. All of these possibilities present the user with new options to consider before planning their final experiment. In addition, the recent development of diffractometers with a smaller sphere of confusion, such as the vertical orientation of the MD3 on the P14 MX beamline at PETRA III, will open the way for users to collect complete data sets for ever smaller crystals by aligning multiple microcrystals. In conclusion, we hope that our results, together with our recent efforts in facilitating such possibilities in *MxCuBE* (Brockhauser *et al.*, 2012 ▶), will prompt all users to take advantage of alignment strategies for their final data collection.

## Figures and Tables

**Table 1:** List of synchrotron-based MX beamlines with MK3 devices

Synchrotron	Beamline
European Synchrotron Radiation Facility (ESRF)	BM14 (http://www.bm14.eu/)
	Structural Biology Beamlines (http://www.esrf.fr/UsersAndScience/Experiments/MX)
	BM30 (http://www.esrf.eu/UsersAndScience/Experiments/CRG/BM30A/)
Advanced Photon Source (APS)	LS-CAT (http://ls-cat.org/index.html)
	NE-CAT (http://necat.chem.cornell.edu)
Diamond Light Source (DLS)	MX beamlines (http://doc.diamond.ac.uk/MXManual)
BESSY	BL14-1 (http://www.helmholtz-berlin.de/forschung/funkma/soft-matter/forschung/bessy-mx/beamlines/bl14-1/index_en.html)
MAX-lab	I911-3 (http://cassiopeia.maxlab.lu.se/index/station-3)
PETRA III	P13 (http://hasylab.desy.de/e70/e231/e33691/p13_macromolecular_crystallography_i/index_eng.html)
	P14 (http://hasylab.desy.de/e70/e231/e33691/p14_macromolecular_crystallography_ii/index_eng.html)
Australian Synchrotron	MX beamlines (http://www.synchrotron.org.au/index.php/aussyncbeamlines/macromolecular-crystallography/beamline-team)
NSRRC Taiwan (NSRRC)	BL15A1 (http://bionsrrc.nsrrc.org.tw/)
Advanced Light Source (ALS)	8.2.1 (http://bcsb.als.lbl.gov/wiki/index.php/8.2.1)
Canadian Light Source (CLS)	08B1-1 (http://cmcf.lightsource.ca/beamlines/08b1-1/)

**Table 2:** Data collection from a bovine trypsin crystal at 13.2keV in several orientations and *SHELXD*/*E* results Values in parentheses are for the highest resolution shell (1.91.8).

Alignment	(0 0 1)	(1 3 1)	(0 1 1)	(2 1 1)
Position of data collection	1	2	2	1
Space group	*P*2_1_2_1_2_1_			
Unit-cell parameters ()	*a* = 54.3, *b* = 58.1, *c* = 66.8			
No. of images	360	360	360	360
()	151.1	148.0	50.6	109.7
()	109.7	146.7	230.4	22.1
Unique reflections	24991 (3560)	25630 (3697)	25614 (3689)	25632 (3698)
Completeness (%)	98.1 (97.3)	100.0 (100.0)	99.9 (99.9)	99.9 (100.0)
Multiplicity†	14.5 [7.7]	14.2 [7.5]	14.2 [7.6]	14.2 [7.5]
*I*/(*I*)	74.8 (35.9)	57.4 (24.4)	65.2 (31.4)	60.0 (31.2)
*R*_meas_ ‡ (%)	2.7 (7.2)	3.5 (9.4)	3.1 (7.8)	3.5 (7.6)
*R*_p.i.m._ § (%)	0.7 (1.9)	0.9 (2.5)	0.8 (2.0)	0.9 (2.0)
*R*_anom_ ¶ (%)	0.8	0.9	0.8	0.9
Anomalous signal to noise††	0.94 (0.84)	0.85 (0.79)	0.85 (0.78)	0.83 (0.76)
*SHELXD*				
FIND/DSUL	14/6	14/6	14/6	14/6
Resolution for *SHELXD* ‡‡ ()	2.3	2.3	2.3	2.3
CC_all_	22.8	16.0	16.0	19.0
CC_weak_	11.0	4.4	5.1	9.3
PATFOM	3.3	2.6	2.9	3.1
*SHELXE*				
Solvent content (%)	57	57	57	57
Contrast	0.52	0.49	0.5	0.59
Connectivity	0.78	0.71	0.71	0.73
Pseudo-free CC (%)	72	55.3	53.75	58.8

† Multiplicity of the native and anomalous (in square brackets) data.

‡ *R*_meas_ = 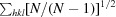 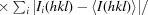 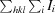 (*hkl*) is the multiplicity (*N*) independent *R*_merge_.

§ *R*_p.i.m._ = 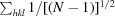 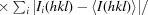 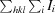 (*hkl*) is a precision-indicating *R* factor.

¶ *R*_anom_ = 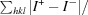 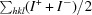 is the ratio of the mean anomalous intensity difference to the mean reflection intensity.

†† Anomalous signal to noise = 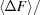 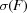 is the ratio of the anomalous difference to the noise.

‡‡ Resolution used for heavy-atom search.

**Table 3:** Data collection and *SHELXD* and *SOLOMON* results for an SeMet-substituted domain from a protein involved in piRNA biogenesis collected in two orientations Values in parentheses are for the highest resolution shell (2.952.80).

Alignment	(0 0 1)	None	Combined
Space group	*P*2_1_2_1_2_1_		
Unit-cell parameters ()	*a* = 35.2, *b* = 100.8, *c* = 146.3		
No. of images	100	100	100 + 100
()	52.0	0	
()	150.01	0	
Unique reflections	13160 (1873)	13576 (1963)	13594 (1948)
Completeness (%)	97.8 (96.9)	99.8 (99.5)	100.0 (99.9)
Multiplicity†	4.0 [2.2]	3.9 [2.1]	7.7 [4.1]
*I*/(*I*)	12.5 (2.4)	10.3 (1.8)	13.7 (2.9)
*R*_meas_ ‡ (%)	14.5 (78.6)	16.7 (106.8)	16.6 (72.7)
*R*_p.i.m._ § (%)	7.1 (38.0)	8.4 (52.8)	5.9 (32.6)
*R*_anom_ [Table-fn tfn10] (%)	10.4	11.7	9.4
Wilson *B* factor (^2^)	62.7	67.2	60.7
Anomalous signal to noise[Table-fn tfn11]	1.35 (0.72)	1.16 (0.68)	1.45 (0.75)
*SHELXD*			
FIND	20	20	20
Resolution for *SHELXD* [Table-fn tfn12] ()	3.3	3.3	3.3
CC_all_	47.9	33.2	50.8
CC_weak_	18.8	7.1	18.9
PATFOM	5.2	3.8	4.9
*SOLOMON* †			
Overall correlation on |*E*|^2^	0.41 *versus* 0.34		0.44 *versus* 0.35

† Multiplicity of the native and anomalous (in square brackets) data.

‡ *R*_meas_ = 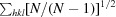 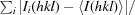 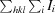 (*hkl*) is the multiplicity (*N*) independent *R*_merge_.

§ *R*_p.i.m._ = 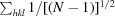 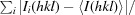 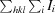 (*hkl*) is a precision-indicating *R* factor.

¶ *R*_anom_ = 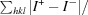 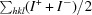 is the ratio of the mean anomalous intensity difference to the mean reflection intensity.

†† Anomalous signal to noise = 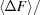 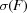 is the ratio of the anomalous difference to the noise.

‡‡ Resolution used for the heavy-atom search.

§§ The correlation coefficient calculated between the structure-factor amplitudes of the ‘observed’ data and the modified map using *SOLOMON* (Abrahams Leslie, 1996 ▶) for the correct *versus* the incorrect hand.
